# Clinical and molecular features and therapeutic perspectives of spinal muscular atrophy with respiratory distress type 1

**DOI:** 10.1111/jcmm.12606

**Published:** 2015-06-20

**Authors:** Fiammetta Vanoli, Paola Rinchetti, Francesca Porro, Valeria Parente, Stefania Corti

**Affiliations:** Dino Ferrari Centre, Neuroscience Section, Department of Pathophysiology and Transplantation (DEPT), University of Milan, Neurology Unit, IRCCS Foundation Ca’ Granda Ospedale Maggiore PoliclinicoMilan, Italy

**Keywords:** spinal muscular atrophy with respiratory distress - SMARD1, motor neuron, therapeutic strategies

## Abstract

Spinal muscular atrophy with respiratory distress (SMARD1) is an autosomal recessive neuromuscular disease caused by mutations in the *IGHMBP2* gene, encoding the immunoglobulin μ-binding protein 2, leading to motor neuron degeneration. It is a rare and fatal disease with an early onset in infancy in the majority of the cases. The main clinical features are muscular atrophy and diaphragmatic palsy, which requires prompt and permanent supportive ventilation. The human disease is recapitulated in the neuromuscular degeneration (*nmd*) mouse. No effective treatment is available yet, but novel therapeutical approaches tested on the *nmd* mouse, such as the use of neurotrophic factors and stem cell therapy, have shown positive effects. Gene therapy demonstrated effectiveness in SMA, being now at the stage of clinical trial in patients and therefore representing a possible treatment for SMARD1 as well. The significant advancement in understanding of both SMARD1 clinical spectrum and molecular mechanisms makes ground for a rapid translation of pre-clinical therapeutic strategies in humans.

## Introduction

Spinal muscular atrophy with respiratory distress type 1 (SMARD1) is a form of SMA with respiratory distress because of diaphragma-tic involvement 1,2. It has been recently renamed distal spinal muscular atrophy type 1 (DSMA1 MIM#604320) and it is also known as hereditary motor neuropathy type 6 (HMN6) 3.

It was first described by Mellins *et al*., in 1974 4 who reported two cases of newborns presenting an atypical variant of Werdnig–Hoffmann’s disease. The main clinical feature was not muscular weakness and hypotonia, as expected, but rather a respiratory distress, secondary to diaphragmatic insufficiency 4. Later Bertini *et al*., in 1989 5 described this disease as a variation in infantile SMA, characterized by a prominent involvement of diaphragm with severe respiratory distress, and it has been recognized as a separate clinical entity only in 1996 6.

SMARD1 seems to be similar to SMA, but it actually differs from a clinical and genetical point of view 3.

Its exact prevalence is not known yet, but studies have shown that diaphragmatic palsy affects approximately 1% of patients diagnosed with early onset SMA 7. Ever since, more than 60 cases have been described 8.

SMARD1 is caused by homozygous or compound heterozygous mutations in the immunoglobulin μ-binding protein 2 (*IGHMBP2*) gene located on chromosome 11q13.2-q13.4 2. Frameshift deletion, inframe deletion, non-sense, splice donor-site and recessive missense mutations have been described 2.

The location and type of mutations do not appear to correlate with the severity of clinical features 9,10.

The result of mutations in *IGHMBP2* gene is a degeneration of motor neurons in the anterior horns, which leads to SMARD1′s main phenotypical features: irreversible diaphragmatic palsy and progressive distal symmetrical muscular weakness (mainly at the lower limb muscles). SMARD1 is considered a fatal form of infantile motor neuron disease.

Life expectancy is very low, with patients dying within 13 months and only few surviving longer 11,12.

## Clinical presentation

### Respiratory distress

Respiratory distress is the main symptom of SMARD1 and it is characterized by being extremely severe and rapidly progressive 3 (Table1). Early signs of the disease are weak cry, inspiratory stridor, trouble eating and recurrent bronchopneumonia. The onset is often very sudden and dramatic, needing prompt and irreversible invasive ventilation. Unfortunately every other surgical attempt to improve the respiratory distress, such as diaphragmatic plication, resulted to be ineffective 3.

**Table 1:** Diagnostic criteria proposed by Pitt *et al*. to allow a more accurate diagnosis of SMARD1 and to help distinguish it from other similar conditions (Pitt et al. 2003)

Clinical criteria	Histopathological criteria	EMG criteria
Low birth weight <3rd percentile	Reduced myelinated fibre diameter in sural nerve biopsies*	Evidence of acute or chronic distal denervation
Onset of symptoms within the first 3 months of life	Slight evidence of progressive myelinated fibre degeneration in biopsies taken up to 3–4 months	Evidence of significant slowing (<70% of LLN) in one or more motor a/o sensory nerves
Unilateral or bilateral diaphragmatic weakness	No evidence of regeneration nor demyelination, that could justify the reduction in fibre size	
Ventilator dependence within <1 month of onset associated to inability to wean		
No evidence of other dysmorphology or other conditions		

* Since the thickness of the myelin sheath is appropriate for the axon size, its reduction in diameter originates from the axon, which size is similarly reduced.

LLN: lower limit of normal range.

The cause of respiratory distress is a diaphragmatic palsy, which usually appears within the first months of life and only in rare cases within the first weeks. It can be seen on a chest X-ray as an abnormal elevation of the dome of diaphragm. The eventration can affect either one or both hemidiaphragms, but it often starts on the right side of the chest, probably because of a pressure practiced on the diaphragm by the liver 1,2,6,7,9.

The eventration of the diaphragm can be very suggestive for SMARD1 when associated with one or more of the following characteristics 7,9,13:

- newborn with respiratory distress, eventually associated with severe bronchopneumonia or any other kind of serious condition (such as near miss sudden infant death syndrome);
- family history of a sudden and inexplicable death of a previous child;
- consanguinity in parents;
- foot and hand muscle weakness and/or distal articular retractions.

### Prenatal signs

Intrauterine growth delay, prematurity and reduced foetal movements can often be associated with SMARD1. These same non-specific signs could be present not only in the affected patients but also in related siblings, where they could eventually be an indication of a form of late onset SMARD1 9.

### Neuromuscular features

Unlike every other patient with SMA, muscular weakness in patients with SMARD1 involves the distal muscles at first and only later the proximal ones 9,13. This can often be accompanied by congenital foot deformations, which can lead to secondary finger contracture, and fatty pads, which are deposits of adipose tissue in the proximal phalanges 9,13. The reasons behind this distal muscular involvement, as well as the precocious involvement of the phrenic nerve are still unknown. It is not clear if this can be related to different susceptibility of specific motor neuron subsets to the reduction in IGHMBP2 protein. Moreover, SMARD1 patients typically present a complete paralysis of the four limbs and later develop a progressive kyphoscoliosis. In 86% of cases, deep tendon reflexes cannot be elicited 9.

### Central, sensory and autonomic nervous system

Advanced SMARD1 often presents a significant autonomic involvement. Symptoms can vary from cardiac arrhythmia, to urinary retention (with need of catheterization), bladder incontinence, excessive sweating and constipation 9,12. Throughout the course of the disease a paralysis of the hypoglossal nerve can also be observed, which can present tongue fasciculations and weakness of the face muscles 3. Like in other motor neuron diseases, in SMARD1 the oculomotor neurons seem to be spared as well 14.

From a neurophysiological point of view, following the diagnostic criteria defined by Pitt *et al*., the EMG must show an acute or chronic distal denervation and a reduction in sensory or motor conduction velocities (<70% of lower limit of normal range). On a sural nerve biopsy, histopathological abnormalities are a decreased size of the myelinated fibres, without signs of regeneration or demyelination 15.

### Juvenile SMARD1

SMARD1 has a considerable variation in onset. In the majority of cases, there is an early onset in infancy, but many cases of juvenile form of SMARD1 have been reported 8,10,13,14,16. To date, the oldest children diagnosed with SMARD1 were in their teen. An exception is represented by a 20-year-old man that developed a diaphragmatic palsy and distal weakness in infancy and retained only facial expressions and small shoulder movements by adulthood. He also had a mild to severe cognitive impairment, which cause still remained unclear, but it might concern the adequacy of ventilation 8,12.

Very recently, Hamilton and his group 17 described a case of a 21-year-old woman affected by uncomplicated SMARD1, that is, able to work and enjoy a social life. She underwent tracheostomy very early in her life and presented a classic distal muscle paralysis. However, she was able to complete her studies at the age of 18 and she is currently working full-time and taking driving lessons using modified motor vehicles.

As for the genetic aspects, the *IGHMBP2* analysis revealed two mutations, one of which has never been described before.

### *IGHMBP2* mutations with a Charcot-Marie-Tooth Disease Type 2 phenotype

Cottenie *et al*., recently investigated an English family with two affected siblings with clinical features consistent with a recessive Charcot-Marie-Tooth type 2 (CMT2), whose genetic analysis revealed compound heterozygous mutations in the *IGHMBP2* gene 18.

The onset of the disease was in the late childhood, with a slow progression: both siblings are still able to work, drive and walk with the assistance of a walking stick and a silicon ankle foot orthosis. Although the younger sister had a clinically milder form, they both presented bilateral foot drop, distal weakness, upper and lower limbs atrophy, absent reflexes, sensory loss in feet and hands, no cranial nerve involvement and a trombone-shaped tongue. Nerve conduction studies, as well as the sural nerve biopsy, demonstrated an axonal neuropathy. Chest X-ray was normal and there were no respiratory problems. This was essentially a typical form of CMT2. Using Exome sequencing, two compound heterozygous mutations in the *IGHMBP2* gene were identified: a non-sense 5′ mutation and a 3′ frameshift mutation in the last exon of the gene, which mother and father were respectively heterozygous for.

After this discovery, *IGHMBP2* was Sanger sequenced in a cohort of 85 recessive CMT2 families and 11 families turned out with *IGHMBP2* mutations. The phenotype was characterized by childhood onset, mild glove and stocking sensory involvement and mild sensory and motor axonal neuropathy, as demonstrated by electrophysiological studies (velocities 40–50 m/sec., in contrast with the severe form of SMARD1 neuropathy). Some cases presented mild scoliosis and other trombone-shaped tongues. None of the cases had respiratory problems nor recurrent airway infections or sleep apnoea.

Unlike patients with *MFN2* mutations, the most frequent form of CMT2, which have a near complete loss of large myelinated fibres and widespread loss of the small ones, patients with *IGHMBP2* mutations have only a reduction in density of the large myelinated fibres, while the small fibres are well preserved.

As for the protein quantification, to establish whether the abundance of residual proteins correlated with the severity of the phenotype, IGHMBP2 was quantified in the fibroblasts and lymphoblasts of patients with *IGHMBP2*-associated CMT2, SMARD1, as well as in carriers and controls. Results demonstrated that patient with CMT2 features had significantly higher IGHMBP2 protein levels than SMARD1, but lower than the controls. This indicates that clinical differences are linked to IGHMBP2 protein levels.

Despite the reduction in protein levels, both SMARD1 and CMT2 had normal *IGHMBP2*-mRNA levels, suggesting that truncated or defective proteins undergo posttranslational degradation.

## IGHMBP2 protein

The *IGHMBP2* gene is composed by 15 exons encoding a protein of 993 amino acids (109,149 D) 19. The Ighmbp2 protein, also called Immunoglobulin S-μ-binding protein 2 (Sμbp-2), has four domains: an ATPase/helicase domain, a single-stranded nucleic acid-binding R3H domain, a DEXDc domain and an AN1-type zinc finger motif 16,19–21. The Ighmbp2 protein is a member of the SF1 helicase, in particular of the Upf1-like subfamily, which differs from the other members thanks to their ability to unwind both DNA and RNA duplex in the 5′→3′ direction 16,22. Its exact function is still unknown, but it might be involved in immunoglobulin class switching, pre-mRNA processing events, regulation of DNA replication or interaction with TATA binding protein 2,23.

The Sμbp-2 protein is ubiquitously expressed and in neurons it is mainly localized in the cytoplasm, as well as in the nucleus, dendrites and axons 24.

Despite the nearly ubiquitous expression of the gene product Ighmbp2 (OMIM*600502), in SMARD1 the α-motoneurons are predominantly affected 19,24.

The first report of *IGHMBP2* gene mutations related to SMARD1 phenotype was published by Grohmann *et al*., in 2001 2. They found three recessive missense mutations, two non-sense mutations, one frameshift deletion and one splice donor mutation in six different SMARD1 families. Moreover, Maystadt *et al*., identified in five SMARD1 patients nine new mutations of this gene, in particular seven missense and two non-sense mutations. Interestingly, seven of those nine mutations were found at highly conserved residues of the supposed DNA helicase domain 25.

As mentioned above, Sμbp-2 has both a RH3 domain and an ATPase/helicase domain. Fukita *et al*., through biochemical analysis, demonstrated that the RH3 domain is involved in the RNA binding process. Even if the N-terminal helicase domain alone is able to bind RNA, it seems that RH3 domain increases this activity, allowing it to operate as an ATP-dependent helicase, binding and unwinding DNA and RNA in a sequence specific manner. In fact, a truncation of the N-terminal sequence of the RH3 domain significantly reduces the binding affinity both with DNA and RNA 20,26.

The RH3 domain could be found in more than 700 proteins, with the same structural sequences Arg-X-X-X-His and in association with other domains, such as DEAH helicase domains, ring-type zinc fingers nuclease domains and ATPase domains. Moreover, in the particular case of Sμbp-2, the segment between 638 and 786 residues where the end R3H C-terminal domain is located, binds ssDNA with 5′phosphorylated guanine rich sequences and it acts as an anchor. The N-terminal domain, on the other hand, interacts with the nucleotides located downstream 20.

Although a genotype–phenotype relation has not been identified yet, it has been observed that seven of the nine typical missense mutations influence the ATPase activity, either through the reduction in the structural stability or through the interference with the ATP binding/hydrolysis of the helicase domain 16.

In particular, the T4931 mutation seems to cause a neuronal degeneration through the reduction in intracellular Sμbp-2 protein levels 23.

In the following years, an increasing number of new mutations in the *IGHMBP2* gene have been described. Twenty-six new mutations in the *IGHMBP2* gene were reported in children who suffered from a severe respiratory distress, because of diaphragmatic paralysis, and from a progressive muscle weakness, which appeared within 6 months of life. Those mutations included fourteen missense, six non-sense, four frameshift, one in-frame deletion and one frameshift insertion 9.

## Molecular features

Currently, little is known about the distribution and role of Ighmbp2 in the pathogenesis of SMARD1. Different types of mutations have been identified in the *IGHMBP2* gene. In particular, in the neuromuscular degeneration (*nmd*) mouse model an aberrant splicing near the 5′ end of the gene has been detected, which comprehends 23 additional nucleotides and a splice donor mutation (A to G) into intron 4, resulting in a premature stop codon. This last mutation interferes with the normal splicing of the mRNA, causing in about 75–80% of cases a mutation splicing and in the other 20–25% a wild-type splicing, not only in the brain and in the spinal cord but also in all other tissues as well 24,27.

As mentioned above, the Ighmbp2 protein is expressed ubiquitously in the organism, but, to better understand its function, it is important to analyse the subcellular localization. For this reason Guenther *et al*., investigated its localization inside the cells, through the use of different α-Ighmbp2 antibodies in primary embryonic mouse motor neurons, which are the first to be affected in SMARD1 16.

The results have shown that Ighmbp2 is mostly localized in the cytoplasm, in particular in the perinuclear cytoplasm, in the axon and in the growth cone. Moreover, in the *nmd* mice it has been observed that the expression of the protein was extremely reduced in all those sites 16.

Another point worth considering, to further comprehend the role of this protein, is which proteins Ighmbp2 is able to bind to. Under this perspective, de Planell-Saguer *et al*., identified Reptin and Pontin, which are two ATPase/helicases proteins, the transcription factor IIIC 220 kD (TFIIIC220) and tubulin, which are able to co-immunoprecipitate with Ighmbp2. Further analysis has shown the real connection between Ighmbp2 and both Reptin and Pontin, as well as with TFIIIC220, but no interaction with tubulin. They also demonstrated the ability of Ighmbp2 to bind itself 24.

Another study of the same group investigated whether Ighmbp2 was also involved in processing, regulation or metabolism of RNA, because of its helicase domain. They observed that Ighmbp2 could bind 3-end of both tRNA^Met^ and tRNA^Arg^ and probably also the 5′-ends of tRNAs. These data suggested that Ighmbp2 was also able to interact with the ribosome, but was not able to correctly translate certain mRNAs, which are important in motor neurons nucleic acid-binding domain 16,24.

Overall, Ighmbp2 acts as an enzyme as well, unwinding RNA and DNA in an ATP-dependent reaction, supporting the theory that it shares not only sequence and structural similarities with the members of UPF1-like helicase within superfamily1, but also its helicase functions 16.

Moreover, Ighmbp2 is also able to hydrolyse ATP and distinguish among NTP co-factors 16.

## Animal models

Animal models are essential to understand the basic mechanisms and the physiology of the diseases. In particular, rodent models, natural and transgenic, provide the opportunity to test the efficacy of potential therapies and to obtain precious information to target treatments and improve gene and drug therapies 28–30.

Different murine models are available for motor neuron diseases (http://www.jax.org/). The model used for SMARD1 is the *nmd* mouse, in particular the B6.BKS *Ighmbp2nmd-2J* mouse 19,24,27,29,31–33.

As in humans, these mice present the *Ighmbp2* region conserved, even if it is located on chromosome 19 instead of chromosome 11 29.

The *nmd* mouse possesses a spontaneous homozygous mutation in the *IGHMBP2* gene and develops a phenotype that is very similar to that of SMARD1 33. The genetic defect consists of a single nucleotide mutation (A-G) into intron 4, causing a reduction in ˜80% of the functional *Ighmbp2* transcript 24,27.

Phenotypically, homozygous mutant mice became distinguishable from their littermate controls by the second week of age. Mutant mice presented dorsally contracted hindlimbs and impaired locomotor activity, and are indeed not able to stand erect 19,27,29,31,33.

The paralysis is followed by the loss of motor neuron innervation and they usually survive only few months 24,27,31,32.

The exact cause of death of affected mice is unclear, but the inability to breathe of some of the end-stage disease animals suggests that the primary cause might be the respiratory failure 27.

## Pathogenesis

So far, it is unknown whether SMARD1 pathology is caused by a defect that primarily interests motor neuron axons and endplates, causing the cell body degeneration, or if it is because of the degeneration of the motor neuron itself, resulting in the loss of the axons and endplates 19.

The *nmd* mouse model can help us to better understand the development of this disease and its pathogenesis.

Different groups have studied the relationship between *Ighmbp2* mRNAs, protein levels and the *nmd* phenotype. Nevertheless, the levels of the protein are reduced by about 20% in *nmd* mice, compared to the control, while the size of the mRNA seems to be normal. It is unclear if the protein is required only in the early-phase of the mammalian development and how the level of Ighmbp2 varies in the spinal cord and motor neuron throughout all stages of life 19.

It has also been described the existence in the murine mammalian genome of a major wild-type modifier locus on chromosome 13 (*Mnm*), that specifically halted motor neuron degeneration and rescued the *nmd* phenotype 27.

The rescue of their phenotype, given by the introduction in transgenic mice of genes and t-RNAs encoded by this region, suggested the existence of genetic modifiers of SMARD1 phenotype that were independent from the rescue of Ighmbp2 level 25.

Another important aspect to consider is the relationship between the time course of the motor neuron degeneration and the onset of clinical symptoms 19. It has been observed that, although at postnatal day 10 the muscular strength in *nmd* mice is still normal, there is already a conspicuous loss of neuron cell bodies in the lumbar spinal cord. This demonstrates that the motor neuron loss is a very early event in this disease, occurring even before the onset of first clinical symptoms 19. A possible hypothesis, supported by neuropathological studies and in accordance with observations in Wallerian degeneration, is that the motor neuron degeneration actually starts at the spinal cord level and then travels along the axon 19. In fact, neuromuscular junction denervation is a subsequent event in the progress of the disease.

Another important aspect to consider is the difference between clinical symptoms in SMARD1 patients and the *nmd* mouse model. First of all, the onset of the diaphragmatic paralysis is an important characteristic of the human disease, which has an early onset, while in *nmd* mice the respiratory distress appears only in the late stages 19. Moreover, in the pathogenesis of *nmd* mice not only is the motor neuron loss involved but also a progressive cardiomyopathy, caused by the death of the cardiomyocytes and by a functional and morphological alteration of the myocardium 31. To investigate these aspects, Maddatu’s group created a transgenic mouse, which expressed full length Ighmbp2 c-DNA only in the central nervous system (CNS), including forebrain, cerebellum and spinal cord. The results showed a sparing of the motor neurons from the degeneration, but the life span was still significantly diminished. In fact, this reduction in life span was because of a congestive heart failure, caused by severe cardiomyocyte degeneration 31.

Subsequently Maddatu’s group created another transgenic mouse, able to produce a wild-type Ighmbp2 both in motor neurons and cardiomyocytes. These mice could not be distinguished from their siblings and the life span was extremely increased, as they lived up to 2 years. Overall these studies showed that the *nmd* mice’s premature death was due also to dilated cardiomyopathy, and not only to motor neuron degeneration 34.

A noteworthy fact is that SMARD1 patients do not develop dilated cardiomyopathy, perhaps because they do not live long enough to develop this kind of symptom 31,34.

## SMARD1 therapy: state of the art and future perspectives

At the present, no effective treatment is available for SMARD1. Diagnosis and symptomatic therapy are delineated during the first hospitalization. Affected children are then usually attended by their families, who have to face a huge care effort. Patients generally require mechanical ventilation 8, antibiotic therapy and prophylaxis for recurrent airway infections. Nutrition is also very important for these patients, because of their feeding difficulty because of muscle weakness and gastrointestinal dysfunction 35. Moreover, physical and occupational therapy are essential aspects of the treatment. Since survival rate is so low, different therapeutic approaches have recently been evaluated, in particular gene and stem cell (SC) therapy.

### Pharmacological treatment

**Figure 1:**
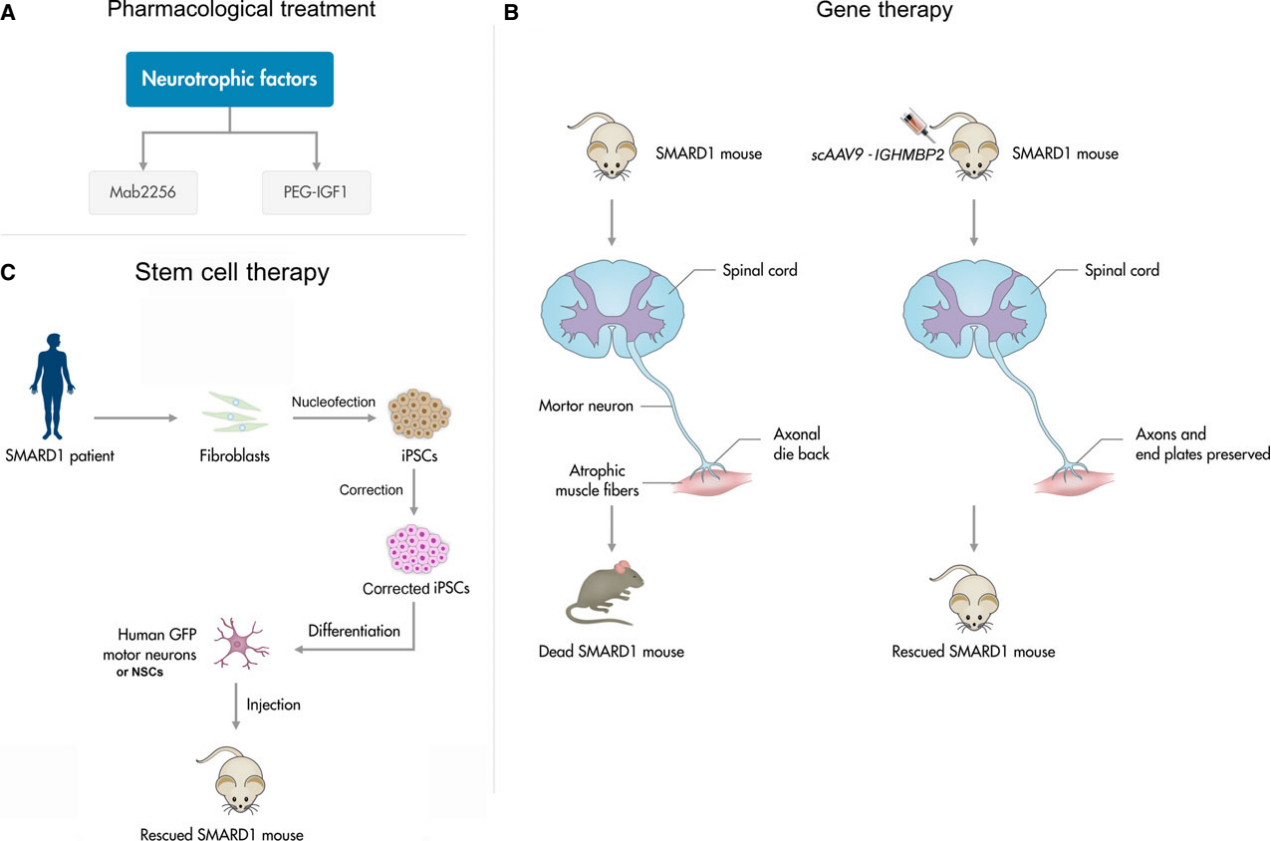
(A) Pharmacological therapeutical approach for spinal muscular atrophy with respiratory distress (SMARD1): Mab2256, a monoclonal antibody with agonist effect on the tyrosine kinase receptor C, implicated in neuron plasticity and synaptic strength; PEG-IGF1, a neurotrophic factor, which low serum levels are likely to be linked with the muscular/neuronal degeneration. (B) Gene-therapy approach: it is based on the replacement of the defective gene, using self-complementary adeno-associated virus vectors. (C) Stem cell-based therapeutic approach: the generation of human induced pluripotent stem cells (iPSCs) can be obtained through the nucleofection of adult fibroblasts with constructs encoding OCT4, SOX2, NANOG, LIN28, c-Myc and KLF4. Uncorrected SMARD1-iPSC-derived motor neurons reproduced disease-specific features, which were ameliorated in motor neurons derived from genetically corrected SMARD1-iPSCs.iPSCs are then differentiated into neural stem cells (NSC) or GFP motorneurons and transplanted into a SMARD1 mouse model, obtaining an improvement in the animal phenotype.

Few pharmacological treatments have been attempted only at the pre-clinical level in SMARD1 (Fig.1A).

Ruiz *et al*., evaluated in the *nmd* mouse model the efficacy of a monoclonal antibody (Mab2256) that has an agonist effect on the tyrosine kinase receptor C (trkC), which is involved in the regulation of neuron plasticity and synaptic strength 33,36. The results showed a significant increase in muscular strength, although only transient, a slowdown in the progression of the disease, but not its stop, and an electrophysiological improvement of the muscular function. However, the survival probability did not show a significant increase 33,36.

A possible explanation of these transient effects could be that high plasma levels of Mab2256 might suppress the expression of trkC, as demonstrated in other studies with neurotrophins (NTs) 37,38. In this case, an adjustment in the dosage of the NTs should avoid this effect. Other possible explanations are a non-sufficient affinity/activity of Mab2256 or that, to have clinical beneficial outcome, more NT receptors have to be activated.

Also neurotrophic factors, such as insulin-like factor 1 (IGF1), have been considered as a possible candidate for future treatment trials, since they seem to play an important role in the pathogenesis of SMARD1, as recently demonstrated by Krieger *et al*., 39. IGF1 is a hormone characterized by numerous functions, like muscle and neuron survival or differentiation and axonal growth during development 39. Krieger *et al*., demonstrated that in the *nmd* model IGF1 serum levels were quite low, supporting the hypothesis of a linkage between IGF1 serum levels and muscular/neuronal degeneration 39. The use of polyethylene glycol-coupled IGF1 (PEG-IGF1) was able not only to restore the endogenous levels of IGF1 but also to reduce the core features of the disease in the *nmd* mouse. This happened through two different mechanisms: (*i*) increased phosphorylation of Akt (protein kinase B) and ribosomal protein S6 kinase, which protects from muscle fibre degeneration and (*ii*) action on sprouting of nerve terminals. However, no significant breakthrough was made in the motor neuron survival. A possible reason for this negative result is that the systemic dose used in this study was not able to reach a sufficient serum concentration to penetrate through the blood–brain barrier, thus making it impossible to reach an effective local tissue concentration.

Based on these findings, IGF1 and other neutrophic factors may hold promise as candidates for future studies in SMARD1 treatment.

### Gene therapy

Gene therapy has the advantage to treat the cause of the disease, allowing the defective gene to be expressed. It is based on the utilization of viral vectors, which carry a healthy copy of the gene into the affected cells (Fig.1B) 40–42. The aim was to correct the aetiological mechanisms underlying the clinical manifestations of the disease 43,44. Many studies have been made in mouse models of SMA, a similar genetic motor neuron disease caused by mutations in *SMN1*, encouraging a future application of gene therapy also in SMARD1.

As for SMA, gene therapy mostly focuses on replacing the defective gene using self-complementary adeno-associated virus vectors 45–48.

In fact, the discovery that the AAV9 vector was able to penetrate the BBB allowed considering the administration of AAV by less-invasive injection, such as systemic intravenous delivery and intrathecal delivery. The administration of AAV9 encoding wild-type *SMN* gene was able to rescue the phenotype of SMA mice when injected intravenously 45,46,48. The biodistribution of AAV in the CNS has been replicated also in larger animals, such as non-human primates 8,18,49. These positive data led to the design and approval, at the Ohio State University, of a Phase I/II clinical trial in SMA patients using intravenous administration and some SMA1 patients have already been treated 50 (www.clinicaltrial.gov).

At the moment, no clinical trial with gene therapy for SMARD1 was planned.

### Stem cell therapy

Currently, SCs represent a promising resource for the treatment of neurodegenerative diseases, such as motor neuron disorders. Stem cells are characterized by their potential to continuously renew themselves by symmetrical division and to originate more mature progenitors of multiple lineages through asymmetrical division 51–53. Neural stem cells (NSCs) are the precursors of the three neuroectodermal lineages in the CNS. Neural stem cells transplantation can provide positive therapeutic effects through multiple mechanisms, including neuroprotection and cell replacement (Fig.1C) 54,55.

Our group previously demonstrated that murine NSC transplanted in a SMARD1 mouse model improved the animal phenotype 56.

In our first set of experiments, we transplanted ALDH high side scatter low (ALDH^hi^SSC^lo^) NSCs in *nmd* mice as a possible therapeutic approach for SMARD1. ALDH^hi^SSC^lo^NSCs cells derived from both embryonic and adult spinal cord and were self-renewing and multipotent. They were able to differentiate into the three main lineages and also to acquire mature complex neural phenotypes, including the expression ofHB9/ChAT in motor neurons, in *nmd* mice both *in vivo* and *in vitro* 56.

More recently, we demonstrated the therapeutic potential of more differentiated cells, such as motor neuron precursors, transplanted into the spinal cord of *nmd* mice 57.

In both cases, using NSCs or more differentiated cells, the positive effects of this approach seemed to derive more from a paracrine effect rather than cell substitution, thanks to the production of neuroprotective factors by induced pluripotent stem cell (iPSC)-derived NSCs, including glial-derived neurotrophic factor, brain-derived neurotrophic factor, transforming growth factor-α and NT3 58. In fact, *in vitro* experiments demonstrated that co-culture of WT human iPSC-derived NSCs protect human SMARD1 iPSC-derived motor neurons against degeneration, probably through the production of NTs 58.

These experiments of NSCs transplantation were performed with primary murine NSCs. The discovery of iPSCs allowed to obtain unlimited human cells, like NSCs, overcoming the necessity to obtain them from CNS.

In a recent study, we demonstrated the potential therapeutical effect that NSCs obtained from human-iPSCs could have in SMARD1. We observed that, after being transplanted into the spinal cord of SMARD1 animal models, NSCs were able to properly engraft and differentiate, giving protection to their endogenous motor neurons and therefore improving their phenotype. To evaluate the effects that NSCs could have in the human disease, we created human SMARD1-iPSCs motor neurons that yet presented a significantly reduced survival and a shorter axon length. The co-culture with healthy wild-type iPSC-NSCs was able to improve the phenotype of the disease and this amelioration can be ascribed both to the production of neurotrophic factors and to the inhibition of Glycogen Synthase Kinase-3 (GSK-3) and HPK1/GCK-like Kinase (HGK). These results prove that the therapeutical use of NSCs derived from pluripotent SCs can be considered as a valid tool to improve SMARD1 phenotype. Another interesting possibility is represented by the possibility to convert the disease phenotype of iPSCs derived from SMARD1 patient in healthy iPSC through an *ex vivo* gene correction and use the corrected cells as potential autologous cell source for transplantation (Fig.1C).

We believe that what would allow to reach a clinically significant level in SMARD1 (and other motor neuron diseases as well) in terms of therapeutic efficacy would be the combining of cell, drug and gene therapy 58.

## Conclusions

Musculoskeletal diseases are the second-greatest cause of disability worldwide 59. Unfortunately, most of these diseases are incurable. Among those, SMARD1 is a devastating neuromuscular disease, which causes infantile death within 13 months of life in the majority of the cases. Only a small number of studies described patients surviving longer 11,60. It is possible indeed that an increased diffusion of next generation sequencing as a diagnostic tool may result in an increased rate of diagnosis, in particular of atypical phenotypes, such as long survivor patients or patients without diaphragmatic involvement.

The pathomechanisms of SMARD1, and in particular the reasons behind the vulnerability in specific motor neuron subsets, is unknown, but addressing this question might allow to define novel therapeutic targets for SMARD1.

Despite the absence of a resolutive therapy, different approaches have been tested, but only at the pre-clinical level, including, pharmacological treatment, gene therapy and cell therapy.

Particularly interesting appears to be the benefits observed with neurotrophic factors, that may warrant further investigation. However, the limit of this approach can be the difficulty in their delivery to CNS as well as the possible systemic side effects.

Gene therapy seems one of the most appealing curative approaches, given their translatability in human. The definition of the therapeutic window in this case will be one of the most important aspects. As for the cellular approach, increasing evidence suggests that NSC transplantation can exert a therapeutic effect on motor neuron disease phenotypes. Our group contributed in studying the utility of this approach in SMARD1. Further studies need to be performed, to define the best cell source, the modality of administration and its safety as well as the extent of the therapeutic impact that can be achieved with this approach.

Combinatory approaches of pharmacological, gene therapy and cellular strategies can be envisioned of the development of effective therapeutic strategies for SMARD1 and other genetic motor neuron diseases.
